# Carabin Deficiency Aggravates Hepatic Ischemia-Reperfusion Injury Through Promoting Neutrophil Trafficking *via* Ras and Calcineurin Signaling

**DOI:** 10.3389/fimmu.2022.773291

**Published:** 2022-02-21

**Authors:** Xuhao Ni, Xiao Wu, Xiao-Xu Zhu, Jian-Hui Li, Xiao-Yu Yin, Ling Lu

**Affiliations:** ^1^ Department of Pancreato-Biliary Surgery, The First Affiliated Hospital of Sun Yat-sen University, Guangzhou, China; ^2^ Hepatobiliary Center, The First Affiliated Hospital of Nanjing Medical University, Nanjing, China

**Keywords:** neutrophil, HIRI, carabin, Ras, calcineurin

## Abstract

Neutrophil infiltration plays an important role in the initial phase of hepatic ischemia and reperfusion injury (HIRI). Despite many different key molecules that have been reported to meditate neutrophil trafficking in HIRI, the mechanism of this process has not been fully elucidated. In this study, we found that Carabin deficiency in myeloid cells (LysMCre : Carabinfl/fl) aggravated IRI-induced hepatic injury and apoptosis through increasing the infiltration of CD11b^+^Ly6G^+^ neutrophils. ImmGen Datasets further revealed that Carabin was expressed in bone marrow neutrophils (GM.BM) but was significantly downregulated in thio-induced peripheral neutrophils (GN.Thio.PC), which was consistently verified by comparing GM.BM and liver-infiltrating neutrophils induced by IRI. Mechanistically, up-regulation of Carabin in GM.BM *in vitro* reduced the expression levels of P-selectin, E-selectin, and αvβ3 integrin through inhibiting Ras-ERK and Calcineurin-NFAT signaling. Furthermore, blocking P-selectin, E-selectin, and αvβ3 integrin in LysMCre : Carabinfl/fl mice decreased the frequency and number of CD11b^+^Ly6G^+^ neutrophils and reversed hepatic ischemia−reperfusion damage. In conclusion, our results provide a new understanding of Carabin, such that it is expressed and functions not only in adaptive immune cells (T and B cells) but also in innate immune cells (neutrophils), contributing to the migration of neutrophils. These findings provide novel and promising therapeutic targets for the prevention of HIRI during liver transplantation or hepatic surgery.

## Introduction

Hepatic ischemia and reperfusion injury (HIRI) mainly results from liver transplantation, liver surgical resection, and hemodynamic shock (1, 2). During the ischemic period, the production of adenosine triphosphate (ATP) is reduced due to insufficient oxygen and the generation of reactive oxygen species (ROS), cytokines, cell-adhesion molecules, and vasoactive agents, which ultimately results in hepatic cell death (3, 4). The shift from ischemia to reperfusion is generally considered to be the main factor leading to liver injury (5, 6). Subsequent reperfusion triggers a cascade of events, including the activation and recruitment of innate and adaptive immune cells, as well as significant liver injury (7). Neutrophil recruitment to the post-ischemic liver is considered to represent a central factor in the pathophysiological and biochemical changes resulting from HIRI (8, 9).

Carabin was firstly identified as a calcineurin- and Ras-binding protein from a yeast two-hybrid screen, and it suppresses CD4^+^ T-cell activation through endogenously interacting with both calcineurin and Ras (10). Carabin has also been reported to be a novel negative regulator of B-cell function in systemic lupus erythematosus (SLE) and B-cell lymphoma through inhibiting the crosstalk between BCR and TLR9 pathways (11, 12). These studies have demonstrated that Carabin is a key regulator involved in the activation of adaptive immune cells. However, its role and underlying mechanisms in innate immune cells remain poorly understood.

In this study, we explored whether Carabin deficiency in myeloid cells (LysMCre : Carabinfl/fl) contributes to HIRI and investigated its underlying signaling mechanisms. We found that LysMCre : Carabinfl/fl mice exhibited dramatically aggravated HIRI, which mainly depended on promoting neutrophil recruitment. Interestingly, we also found that Carabin expression was downregulated in neutrophils when stimulated by thioglycollate and induced by IRI. The overexpression of Carabin in neutrophils inactivated Ras-ERK and calcineurin-NFAT pathways and decreased the expression levels of P-selectin, E-selectin, and αvβ3 integrin. Moreover, the blockage of P-selectin, E-selectin, and αvβ3 integrin in LysMCre : Carabinfl/fl mice reversed neutrophil recruitment and alleviated hepatic ischemia/reperfusion (I/R) damage.

## Results

### Carabin Deficiency in Myeloid Cells Aggravates HIRI

**Figure 1:**
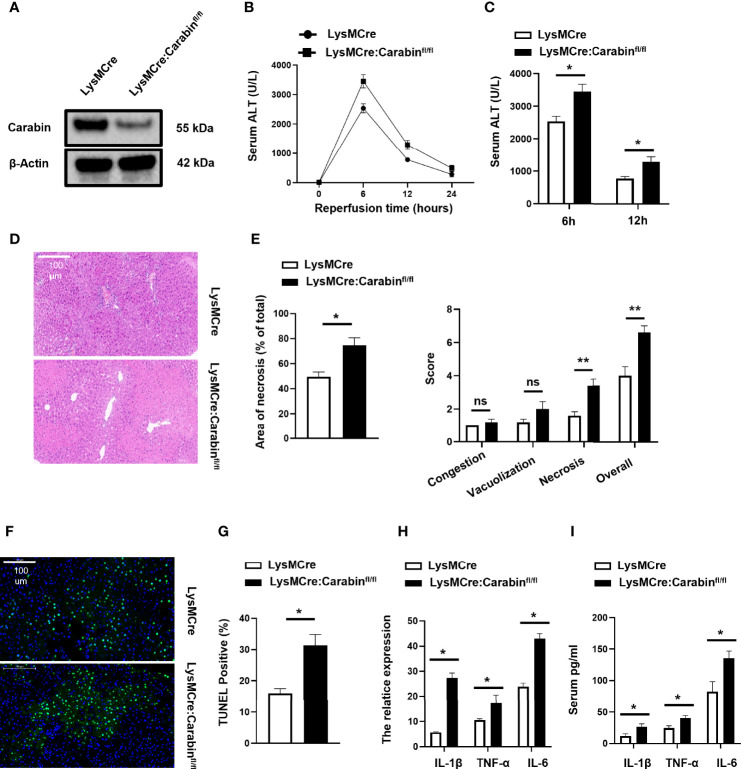
Carabin deficiency aggravates liver damage following hepatic I/R. **(A)** CD11b^+^ cells from Carabinfl/fl, LysMCre and LysMCre: Carabinfl/fl transgenic mice were isolated by flow cytometry, and Western-blot analysis for Carabin was performed. **(B)** Serum ALT levels in LysMCre and LysMCre: Carabinfl/fl mice were detected at 0, 6, 12, and 24 h after reperfusion. Data are presented as the mean ± SEM (n = 5 mice/group). **(C)** Analysis of serum ALT levels in LysMCre and LysMCre : Carabinfl/fl mice subjected to reperfusion for 6 and 12 h. **(D)** H&E staining of liver tissue in each treatment group was performed to evaluate hepatic damage. Scale bar, 100 µm. **(E)** Statistical analyses of the area of necrosis. Data are presented as mean ± SEM (n = 5 mice/group, 3-5 fields were quantified). **(F)** Hepatic apoptosis was detected by TUNEL assays. Scale bar, 100 µm. **(G)** Statistical analyses of the number of TUNEL-positive cells. Data are presented as the mean ± SEM (n = 5 mice/group, 3-5 fields were quantified). **(H)** Expression levels of IL-1β, TNF-α, and IL-6 were measured by qRT-PCR. Data are presented as mean ± SEM (n = 5 mice/group). **(I)** Serum IL-1β, TNF-α, and IL-6 levels were measured by ELISAs. Data are presented as mean ± SEM (n = 5 mice/group). *P < 0.05; **P < 0.01. ns, no significant.

To investigate the potential role of Carabin in the biology of innate immune cells, we first crossed Carabinfl/fl mice to LysMCre transgenic mice to generate mice with myeloid-cell-specific deletion of Carabin (LysMCre : Carabinfl/fl). We isolated myeloid cells from bone marrow and measured Carabin expression *via* Western blotting. The data verified that carabin was deleted in myeloid cells in LysMCre : Carabinfl/fl mice (**Figure 1A**).

In order to dissect the contribution of LysMCre : Carabinfl/fl mice to HIRI, we performed partial (70%) warm hepatic I/R in myeloid-cell-specific Carabin knockout mice (LysMCre : Carabinfl/fl), control mice (LysMCre), and sham-treated mice. Serum ALT levels were monitored at 0, 6, 12, and 24 h after reperfusion. We found that serum ALT levels were significantly increased in LysMCre : Carabinfl/fl mice compared with those in LysMCre mice (**Figures 1B, C**). To further determine the critical role of Carabin in liver injury, we performed H&E staining of liver tissue with different mice and revealed that hepatocyte necrosis in LysMCre : Carabinfl/fl mice was markedly severe in LysMCre mice (**Figure 1D**). The histological examination further demonstrated significantly increased necrotic areas and suzuki’s score in LysMCre : Carabinfl/fl mice compared to WT controls (**Figure 1E**). We also performed TUNEL assays to analyze hepatic cell apoptosis, which revealed that the frequency of hepatocyte apoptosis was significantly increased in LysMCre : Carabinfl/fl mice compared with that in LysMCre mice (**Figures 1F, G**).

Meanwhile, the expression and secretion of TNF-α, IL-1β, and IL-6 were measured. Compared with those in LysMCre mice, the mRNA and serum levels of TNF-α, IL-1β, and IL-6 were all significantly increased in LysMCre : Carabinfl/fl mice after HIRI (**Figures 1H, I**). These results revealed that Carabin deficiency in myeloid cells aggravated HIRI, which might be associated with proinflammatory cytokines.

### Carabin Deficiency Promotes Neutrophil Recruitment in HIRI

**Figure 2:**
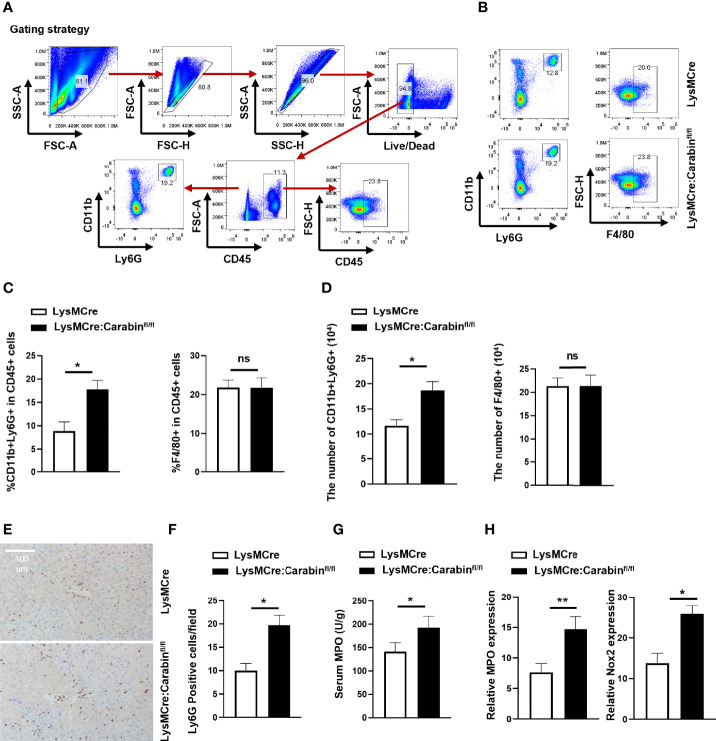
Knockdown of Carabin in myeloid cells increases I/R-induced neutrophil infiltration. **(A)** Strategy for analyzing mature macrophages, Kupffer cells (KC) (F4/80^+^), and granulocytes (CD11b^+^Ly6G^+^) in liver-infiltrating leukocytes *via* flow cytometry. **(B)** Flow cytometry was used to measure Kupffer cells (KC) (F4/80^+^) and granulocytes (CD11b^+^Ly6G^+^) in liver-infiltrating leukocytes. **(C)** Statistical analyses of percentages of CD11b^+^Ly6G^+^ and F4/80^+^ CD45^+^ cells in liver-infiltrating leukocytes. Data are presented as mean ± SEM (n = 5 mice/group). **(D)** Statistical analyses of the numbers of CD11b^+^Ly6G^+^ and F4/80^+^ of CD45^+^ cells in liver-infiltrating leukocytes. Data are presented as mean ± SEM (n = 5 mice/group). **(E)** Ly6G-positive cells in liver tissue were detected by immunohistochemistry. Scale bar, 100 µm. **(F)** Statistical analyses of the number of Ly6G-positive cells. Data are presented as the mean ± SEM (n = 5 mice/group, 3-5 fields were quantified). **(G)** Serum MPO levels were measured by ELISAs. Data are presented as mean ± SEM (n = 5 mice/group). **(H)** The expression levels of MPO and Nox2 were measured by qRT-PCR. Data are presented as means ± SEM (n = 5 mice/group). Data are presented as means ± SEM (n = 5 mice/group). *P < 0.05; **P < 0.01. ns, no significant.

Previous data have suggested that target genes can be effectively deleted in 83%–98% of mature macrophages and nearly 100% of granulocytes in double-mutant mice harboring both the LysMcre allele and one of two different loxP-flanked alleles (13). For these reasons, we next analyzed the frequencies of mature macrophages, Kupffer cells (KC) (F4/80^+^), and granulocytes (CD11b^+^Ly6G^+^) in liver-infiltrating leukocytes *via* flow cytometry at 6 h after reperfusion. The relative proportions of CD11b^+^Ly6G^+^ neutrophils among liver-infiltrating leukocytes in LysMCre : Carabinfl/fl mice were increased compared to those in LysMCre mice (**Figures 2A, B**). However, there was no significant difference in the frequency of F4/80^+^ cells between these two groups (**Figure 2C**). Meanwhile, we counted the numbers of CD11b^+^Ly6G^+^ neutrophils and F4/80^+^ cells in the liver and found that LysMCre : Carabinfl/fl mice had more CD11b^+^Ly6G^+^ neutrophils than did LysMCre mice, whereas there was no difference in the number of F4/80^+^ cells between these two groups (**Figures 2D, E**). To further verify the role of Carabin in neutrophil recruitment, Ly6G and F4/80 IHC was performed to detect Ly6G and F4/80-positive cell populations in liver tissue at 6 h after reperfusion, the findings of which were consistent with those of flow cytometry (**Figure 2F** and **Supplementary Figure 1A**).

Meanwhile, LysMCre : Carabinfl/fl mice displayed higher serum myeloperoxidase (MPO) levels and higher mRNA levels of MPO and Nox2 at 6 h after HIRI compared with those in LysMCre mice (**Figures 2G, H**). Collectively, these results suggested that Carabin deficiency in myeloid cells aggravated HIRI by promoting neutrophil infiltration but not affecting mature macrophages.

### Carabin Regulates the Expression Levels of P-Selectin, E-Selectin, and αvβ3 Integrin In Neutrophils Through Inhibiting Ras-ERK and Calcineurin-NAFT Pathways

**Figure 3:**
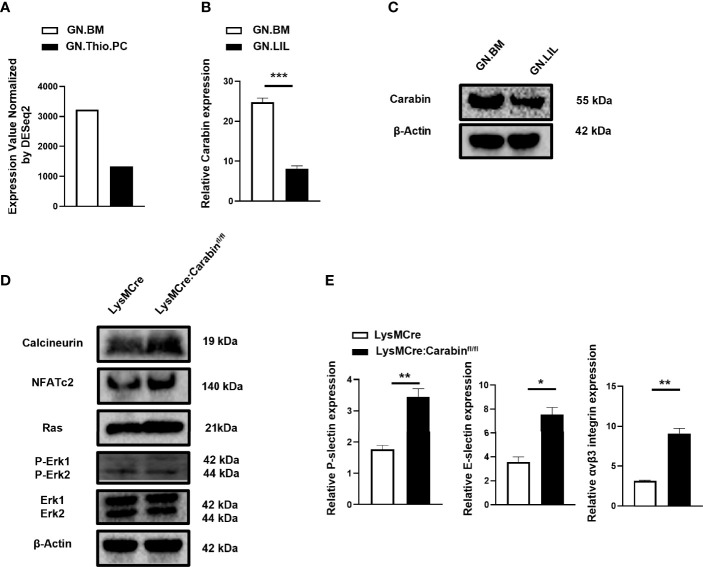
Carabin suppresses the expression levels of P-selectin, E-selectin, and αvβ3 integrin in neutrophils through inhibiting Ras-ERK and Calcineurin-NAFT pathways. **(A)** The mRNA level of Carabin was analyzed from ImmGen datasets in thio-induced peripheral neutrophils (GN.Thio.PC) and bone marrow neutrophils (GM.BM). **(B)** The mRNA level of Carabin was measured in CD11b^+^Ly6G^+^ neutrophils isolated from liver-infiltrating leukocytes after HIRI in WT mice and in bone marrow cells in sham-control mice. **(C)** Western blotting was used to test the protein expression of Carabin in CD11b^+^Ly6G^+^ neutrophils isolated from liver-infiltrating leukocytes after HIRI in WT mice and in bone marrow cells in sham control mice. **(D)** The protein levels of calcineurin, NFAT, Ras, ERK, and P-ERK were measured by Western blotting in liver-infiltrating CD11b^+^Ly6G^+^ neutrophils isolated from LysMCre : Carabinfl/fl mice and LysMCre mice after HIRI. **(E)** The expression levels of P-selectin, E-selectin, and αvβ3 integrin were measured by qRT-PCR. Data are presented as the mean ± SEM (n = 5 mice/group). Data are presented as mean ± SEM (n = 5 mice/group). *P < 0.05; **P < 0.01; ***P < 0.001.

In order to further clarify whether the expression of Carabin changes during the migration of neutrophils, we first found that the mRNA expression of Carabin was lower in thio-induced peripheral neutrophils (GN.Thio.PC) than in bone marrow neutrophils (GM.BM) from ImmGen datasets (**Figure 3A**). In addition, the CD11b^+^Ly6G^+^ neutrophils isolated from liver-infiltrating leukocytes after HIRI in WT mice displayed lower mRNA and protein levels of Carabin compared with those in GM.BM isolated from bone marrow cells in sham-operated mice (**Figures 3B, C**). These data corroborated our earlier discovery that Carabin played a critical role in neutrophil infiltration.

Carabin had been reported to play a key role in T- and B-cell activation through regulating Ras and calcineurin pathways (10–12). To gain further insight into the mechanism by which Carabin contributes to neutrophil migration, we isolated CD11b^+^Ly6G^+^ neutrophilic liver-infiltrating leukocytes at 6 h after HIRI in LysMCre : Carabinfl/fl mice and LysMCre mice and measured the protein levels of calcineurin, NFAT, Ras, ERK, and P-ERK *via* Western blotting. Interestingly, the protein levels of calcineurin, NFAT, Ras, and P-ERK in LysMCre : Carabinfl/fl mice were similar to those in LysMCre mice (**Figure 3D**).

Calcineurin-NFAT and Ras-ERK pathways have been reported to be involved in neutrophil migration by regulating cell-adhesion molecule expression (P-selectin, E-selectin, αvβ3 integrin) (14–19). For this reason, we further determined the mRNA levels of P-selectin, E-selectin, and αvβ3 integrin. Inconsistently, their mRNA levels were significantly increased in LysMCre : Carabinfl/fl mice compared with those in LysMCre mice (**Figure 3E**). We therefore postulated that Carabin-mediated down-regulation of P-selectin, E-selectin, and αvβ3 integrin through inhibiting Ras-ERK and Calcineurin-NFAT signaling may contribute to neutrophil recruitment and HIRI.

### Blockage of P-Selectin, E-Selectin, and αvβ3 Integrin Rescues Carabin-Induced Exacerbation of HIRI

**Figure 4:**
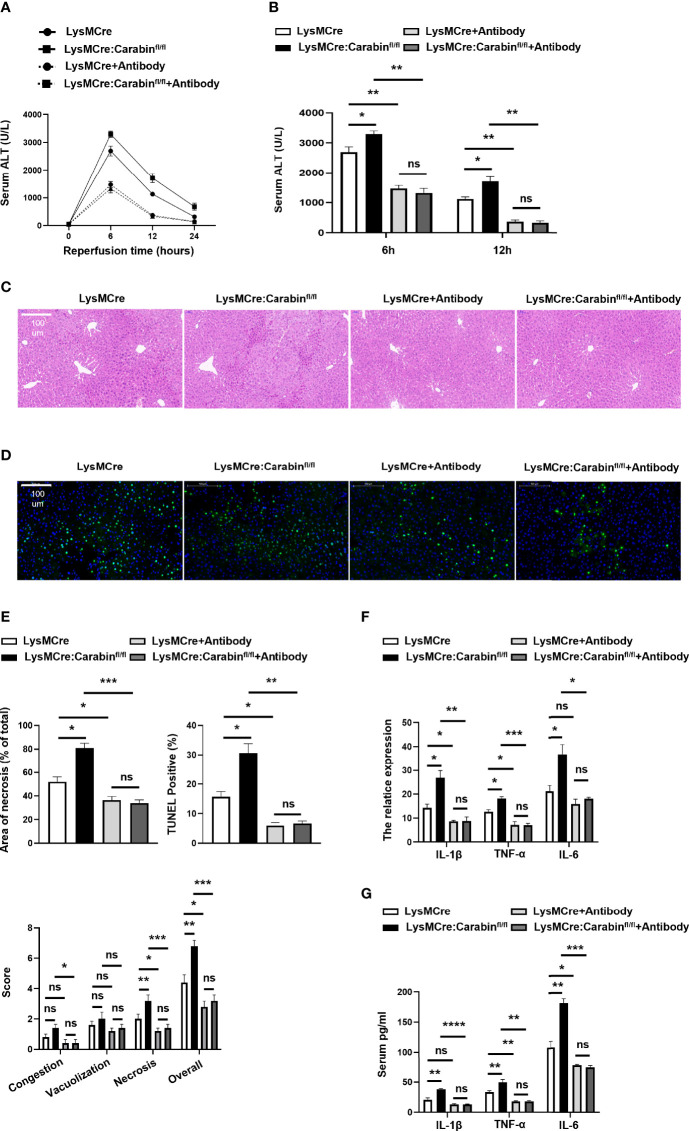
Blocking P-selectin, E-selectin, and αvβ3 integrin alleviates the inflammation response pathway. **(A)** Serum ALT levels in LysMCre and LysMCre : Carabinfl/fl mice with or without P-selectin, E-selectin, and αvβ3 integrin antibody treatments were detected at 0, 6, 12, and 24 h after reperfusion. Data are presented as mean ± SEM (n = 5 mice/group). **(B)** Analysis of serum ALT levels in LysMCre and LysMCre : Carabinfl/fl mice with or without P-selectin, E-selectin, and αvβ3 integrin antibody treatments subjected to reperfusion for 6 and 12 h. **(C)** Representative H&E staining images of hepatic samples in LysMCre and LysMCre : Carabinfl/fl mice in the presence of absence of P-selectin, E-selectin, and αvβ3 integrin antibody treatments after HIRI. Scale bar, 100 mm. **(D)** Hepatic apoptosis of LysMCre and LysMCre : Carabinfl/fl mice treated with or without P-selectin, E-selectin, and αvβ3 integrin antibodies after HIRI was detected by TUNEL assays. Scale bar, 100 µm. **(E)** Statistical analyses of the area of necrosis and the number of TUNEL-positive cells. Data are presented as mean ± SEM (n= 5 mice/group, 3-5 fields were quantified). **(F)** The mRNA levels of IL-1β, TNF-α, and IL-6 were measured by qRT-PCR. Data are presented as mean ± SEM (n = 5 mice/group). **(G)** Serum IL-1β, TNF-α, and IL-6 levels were measured by ELISAs. Data are presented as mean ± SEM (n = 5 mice/group). *P < 0.05; **P < 0.01; ***P < 0.001. ns, not significant.

To explore the involvement of P-selectin, E-selectin, and αvβ3 integrin in the Carabin-deficient driving of neutrophil recruitment after HIRI, the effects of P-selectin, E-selectin, and αvβ3 integrin antibody treatments in an *in-vivo* hepatic I/R model were investigated. As expected, blocking P-selectin, E-selectin, and αvβ3 integrin greatly alleviated the degree of liver injury both in LysMCre : Carabinfl/fl mice and LysMCre mice. Combined treatment with P-selectin, E-selectin, and αvβ3 integrin antibodies significantly decreased serum ALT levels in LysMCre : Carabinfl/fl mice to an extent similar to that in LysMCre mice (**Figures 4A, B**). H&E staining and TUNEL IHC revealed similar trends (**Figures 4C–E**). Consistently, serum TNF-α, IL-1β, and IL-6 levels and tissue TNF-α, IL-1β, and IL-6 mRNA levels in LysMCre : Carabinfl/fl mice and LysMCre mice with P-selectin, E-selectin, and αvβ3 integrin antibody treatments were lower than those in counterparts not treated with an antibody (**Figures 4F, G**). These results suggest that P-selectin, E-selectin, and αvβ3 integrin were responsible for the role of Carabin in HIRI.

### Blocking P-Selectin, E-Selectin, and αvβ3 Integrin Reverse the Influence of Carabin in Neutrophil Recruitment

**Figure 5:**
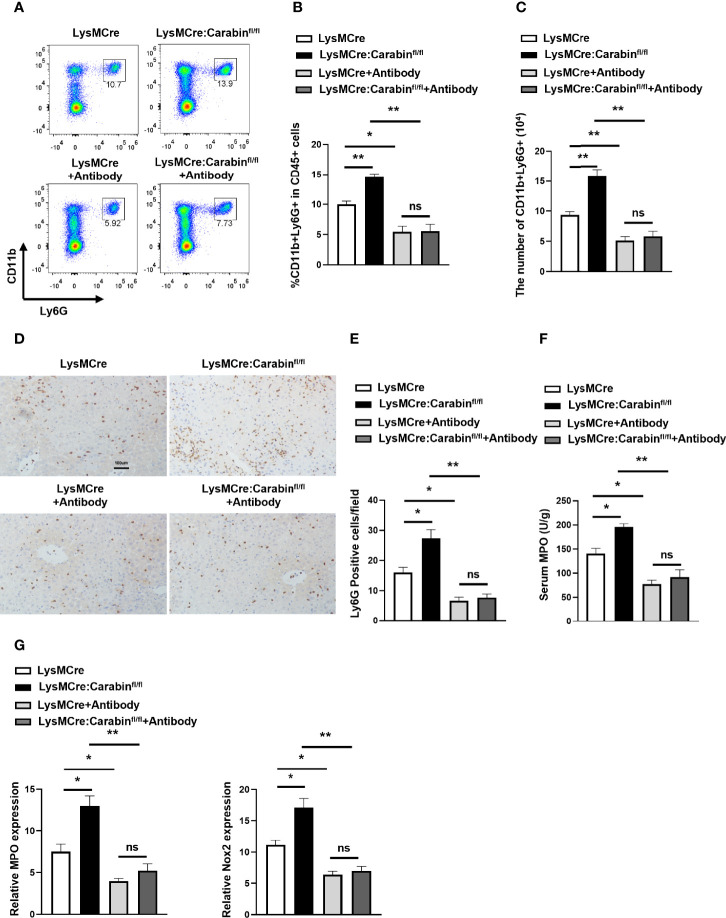
Carabin alleviates hepatic I/R injury through inhibiting the expression of P-selectin, E-selectin, and αvβ3 integrin. **(A)** Flow cytometry measuring granulocytes (CD11b^+^Ly6G^+^) in liver-infiltrating leukocytes. **(B)** Statistical analysis of the percentage of CD11b^+^Ly6G^+^ cells in liver-infiltrating leukocytes. Data are presented as mean ± SEM (n = 5 mice/group). **(C)** Statistical analyses of the number of CD11b^+^Ly6G^+^ cells in liver-infiltrating leukocytes. Data are presented as mean ± SEM (n= 5 mice/group). **(D)** Ly6G-positive cells in liver tissues were detected by immunohistochemistry. Scale bar, 100 µm. **(E)** Statistical analyses of the number of Ly6G-positive cells. Data are presented as mean ± SEM (n= 5 mice/group, 3-5 fields were quantified). **(F)** Serum MPO levels were measured by ELISAs. Data are presented as mean ± SEM (n = 5 mice/group). **(G)** The mRNA levels of MPO and Nox2 were measured by qRT-PCR. Data are presented as mean ± SEM (n = 5 mice/group). Data are presented as mean ± SEM (n = 5 mice/group). *P < 0.05; **P < 0.01; ns, not significance.

Depending on our notion that Carabin deficiency facilitates neutrophils recruitment through P-selectin, E-selectin and αvβ3 integrin, we determined whether the promotion of neutrophil recruitment seen in LysMCre : Carabinfl/fl mice could be restored by P-selectin, E-selectin, and αvβ3 integrin antibodies. The results from flow cytometry showed that the frequency of CD11b^+^Ly6G^+^ neutrophils in the liver was markedly decreased in groups treated with antibodies compared with that in the untreated group (**Figures 5A, B**). The number of CD11b^+^Ly6G^+^ neutrophils in the liver showed a similar trend (**Figure 5C**). Meanwhile, we further tested Ly6G-positive cells in liver tissue *via* IHC. As expected, treatments with P-selectin, E-selectin, and αvβ3 integrin antibodies resulted in a reduction of Ly6G-positive cells in LysMCre : Carabinfl/fl mice that was comparable to the level in LysMCre mice (**Figures 5D, E**). Ly6G-positive cells in LysMCre : Carabinfl/fl mice and LysMCre mice was no significant (**sFigure 1B**). Anti-P-selectin, E-selectin, and αvβ3 integrin treatment also successfully reduced serum MPO levels and mRNA levels of MPO and Nox2 to the same levels as those in LysMCre : Carabinfl/fl mice and LysMCre mice (**Figure 5F, G**). Collectively, our findings support the conclusion that Carabin deficiency can promote neutrophil recruitment in HIRI by increasing P-selectin, E-selectin, and αvβ3 integrin expression.

## Discussion

HIRI is an intense inflammatory and pathophysiological process that results in increased liver injury and is directly associated with the morbidity and mortality of patients receiving liver transplantations, liver surgical resections, and those suffering from hemodynamic shock (20). Reducing hepatic inflammation has been reported to play a pivotal role in preventing liver injury and restoring liver function during HIRI (21, 22). The release of endogenous molecules such as danger-associated molecular patterns (DAMPs) and the production of ROS, cytokines, and other agents during ischemia activate Kupffer cells and stimulate them to produce ROS and proinflammatory cytokines at the early stage of reperfusion (3, 4, 23). Many studies have demonstrated that this process triggered by Kupffer cells further leads to the activation and migration of neutrophils, which are central factors in the pathophysiological and biochemical changes resulting from HIRI (24–26).

In this study, we demonstrated that Carabin deficiency in myeloid cells aggravated IRI-induced hepatic injury and apoptosis by promoting the infiltration of neutrophils into the inflammatory microenvironment of hepatic tissue and enhanced inflammatory responses and oxidative stress. Carabin was first determined to be an endogenous negative-feedback inhibitor of Calcineurin and Ras T-cell activation (10). Previous studies have shown that Carabin inhibits the calcineurin-NFAT signaling pathway and blocks the Ras-ERK pathway through its C-terminal carboxy-terminal domain and N-terminal Ras GTPase-activating protein (GAP) domain, respectively (10). Carabin has also been demonstrated to be a negative regulator in B-cell activation in SLE and B-cell lymphoma through inhibiting crosstalk between BCR and TLR9 pathways (11, 12). Taken together, these studies suggest that Carabin might be a key negative regulator in the activation of the immune system.

More importantly, in our present study, we further revealed that the expression of Carabin in neutrophils was vigorously downregulated by inducing thioglycolate (TGA) and hepatic I/R injury. These results strongly suggest that Carabin might be a negative regulator in the migratory function of neutrophils in a sterile inflammatory microenvironment.

Previous studies have demonstrated that Calcineurin plays a crucial role in the migration of neutrophils through αvβ3 integrin distributions (15–17). A recent study identified that dexamethasone could prolong the expression of αvβ3 integrin and upregulate the expression of the αvβ3 integrin subunit through the calcineurin/NFAT pathway (14). Da-Long. et al. reported that melatonin, a major sleep-adjusting hormone, inhibited neutrophil migration through suppressing the expression of cell-adhesion molecule expression (e.g., P-selectin, E-selectin) regulated by ERK activation (18). Interestingly, the degree of ERK activation is a graded rather than an all-or-none phenomenon in the process of neutrophil chemotaxis toward the bacterial peptide, N-formyl-Met-Leu-Phe (fMLP), which implies that ERK activation might be precisely regulated (19). In our present study, we supported that Carabin deficiency enhanced the activation of calcineurin-NFAT and Ras-ERK pathways during the process of neutrophil migration, which were consistent with the upregulation of P-selectin, E-selectin, and αvβ3 integrin. Consistent with these results, the blockage of P-selectin, E-selectin, and αvβ3 integrin reversed the effect of Carabin deficiency on neutrophil migration and HIRI. Based on our data, we identified the mechanisms by which Carabin in neutrophils may be involved in HIRI.

In conclusion, our study identified that Carabin, as a pivotal negative regulator, is expressed and functions not only in adaptive immune cells (T and B cells) but also in innate immune cells (neutrophils), and was associated with neutrophil migration and hepatic I/R injury. These findings provide novel and promising therapeutic targets for the prevention of HIRI during liver transplantation or hepatic surgery.

## Materials and Methods

### Animals

We generated Carabin flox/flox (Carabinfl/fl) mice on a C57BL/6 background in the Model Animal Research Center of Nanjing University. LysMCre transgenic mice were purchased from the Model Animal Research Center of Nanjing University. These mice were crossed to generate mice specifically lacking Carabin in myeloid cells (LysMCre : Carabinfl/fl) in the same center. All of the mice were housed in a specific pathogen-free facility in accordance with the guidelines of our institution.

### Mouse Model of Liver I/R and Administration of Blocking Antibodies

Partial (70%) warm hepatic I/R in mice was performed as previously described (27). After 60 min of liver ischemia by vascular occlusion, the clip was carefully removed, and mice were reperfused and euthanized immediately at 0, 6, 12, or 24 h post-reperfusion. Blood and liver tissue samples were collected from anesthetized mice and were then preserved in liquid nitrogen for further phenotypic analyses. Sham controls were subjected to the same operation procedure but without clamping. For antibody treatment, approximately 1 h before hepatic I/R, mice were administered with isotype antibody (A110-1, BD Biosciences, San Jose city, CA, USA) (800 ug) or P-selectin (RB40.34, Thermo Scientific, Waltham, MA, USA) (200 ug), E-selectin (BE0294, BioXcell, Lebanon, NH, USA) (200 ug), and αvβ3 integrin antibody (RMV-7/2C9.G2, Biolegend, San Diego, CA, USA) (200/200 ug) *via* intraperitoneal injection(s).

### Measurement of Aminotransferase Levels

Blood samples from each group were obtained from anesthetized mice at 0, 6, 12, and 24 h after reperfusion, after which the serum was collected by centrifugation at 3,000 rpm for 10 min at 4°C. Alanine aminotransferase (ALT) levels in serum were measured using an Alanine Aminotransferase Assay Kit (C009-2, Nanjing Jiancheng Bioengineering Institute, Nanjing, China) according to the manufacturer’s instructions.

### Hematoxylin and Eosin Staining and Immunohistochemistry of Liver Tissues

Liver tissue from each group was fixed using 4% paraformaldehyde (PFA) and was then embedded in paraffin for 24 h. The sections were cut at a thickness of 4 mm and were stained with hematoxylin and eosin (H&E) for histological examinations. Suzuki’s score, consisting of three aspects (hepatocyte necrosis, sinusoidal congestion, and ballooning degeneration), was rated on a scale of 1 to 4. Immunohistochemistry (IHC) was performed to measure the number of Ly6G-positive cells and F4/80-positive cells in the liver, as previously described (28). In brief, the sample sections were routinely deparaffinized and rehydrated, followed by antigen retrieval using 10 mM of sodium citrate buffer (pH 6.0). After blocking with 5% bovine serum albumin, the slides were incubated with Ly6G primary antibodies (Cat#ab238132, Abcam, Cambridge, UK) (1:100) and F4/80 primary antibodies (Cat#ab111101, Abcam) (1:100) at 4°C overnight. Then, the sections were incubated with a secondary antibody (1:200) at room temperature for 30 min, followed by staining with 3,39-diaminobenzidine (Agilent, Santa Clara, CA, USA) and counterstaining with H&E. Positive-Ly6G cells and F4/80 cells in the images were quantified using Image-Pro Plus software (version 6.0).

### Terminal Deoxynucleotidyl Transferase dUTP Nick-End Labeling Assay

Terminal deoxynucleotidyl transferase dUTP nick-end labeling (TUNEL) assays were used to assess apoptosis *via* the *In Situ* Cell Death Detection Kit (11684795910; Roche, Basel, Switzerland), following the manufacturer’s instructions. Images of TUNEL-positive cells were captured by fluorescent microscopy (DMi8; Leica Microsystems, Wetzlar, Germany), we counted the number of TUNEL-positive cells per section (×100um) using a quantitative digital analysis imaging system, Image-Pro Plus (version 6.0).

### Quantitative Real-Time PCR

RNA was extracted using Trizol (Invitrogen, Waltham, MA, USA), followed by cDNA synthesis using SuperScript III (Invitrogen) in a 20-µl reaction volume/well. The same amount of RNA was used in each cDNA synthesis reaction measured by a NanoDrop Spectrophotometer (Thermo Scientific). The same volume of cDNA per sample was prepared for real-time PCR analysis using SYBR Green (Thermo Scientific) and the indicated primers (IL-1β, TNF-α, IL-6, MPO, Nox2, P-selectin, E-selectin, and αvβ3 integrin) to assess transcript levels of each gene. Triplicate reactions were run using an ABI Prism 7500 (Thermo Scientific). mRNA levels were determined by comparative CT method and normalized to β-Actin expression. Primer sequences used in this study for PCR amplification were listed in **Supplementary Materials**.

### Enzyme-Linked Immunosorbent Assays

Enzyme-linked immunosorbent assays (ELISAs) were performed to determine the serum concentrations of cytokines (IL-1β, TNF-α, and IL-6) and MPO *via* a Multiplex ELISA kit (R&D Systems, Minneapolis, MN, USA), according to the manufacturer’s protocol. Fluorescence intensity was quantified and analyzed using a Q-Analyzer (RayBiotech, Norcross, GA, USA).

### Flow Cytometry Analysis

Lymphocytes isolated from livers by percoll (Sigma, St. Louis, MO, USA) were washed three times in phosphate-buffered saline (PBS) containing 1% fetal bovine serum (FBS; FACS Buffer). For detecting dead cells, harvested cells were stained *via* a LIVE/DEAD Fixable Far-Red-Dead Cell Stain Kit (1:1000) using either 633 or 635 nm excitation (Cat#L34973, Thermo Fisher Scientific). For Brilliant Violet 510-CD45 (Cat#103137, BioLegend) (1:400), Alexa Fluor 700-CD11b (Cat#101222, BioLegend) (1:400), PerCP/Cyanine5.5-Ly6G (Cat#127616, BioLegend) (1:600), and PE/Cyanine7-F4/80 (Cat#123114, BioLegend) (1:400) staining, harvested cells were washed and incubated in PBS containing 1% FBS containing fluorochrome-conjugated antibodies in a U-bottom 96-well plate. The frequency of the different cell population was measured *via* flow cytometry (BD LSRFortessa, BD Biosciences) and analyzed by FlowJo_v10 (BD, Ashland, OR, USA). Antibodies used in this study for FC were listed in **Supplementary Materials**.

### Neutrophil Isolation

Liver-infiltrating leukocytes were recovered by percoll (Sigma) gradient centrifugation. Bone marrow cells were harvested after removing red blood cells *via* ACK buffer (Thermo Scientific). CD45^+^CD11b^+^Ly6G^+^ neutrophils were stained and sorted from the liver and bone marrow by using a FACSAria II cell sorter (BD Biosciences, Franklin Lakes, NJ, USA).

### Western Blotting

For Western blotting experiments, cells were lysed in RIPA buffer containing 50 mM of Tris/HCl, (pH of 7.4) with1% Nonidet P-40, 0.5% Nadeoxycholate, 150 mM of NaCl, 1 mM of EDTA, 1 mM of PMSF, 1 mM of Na3VO4, 1 mM of NaF, and protease inhibitor (Sigma). Then cells were separated *via* SDS/PAGE, followed by being transferred to a 0.45-mm-pore PVDF membrane (MilliporeSigma, Burlington, MA, USA). Later, the membrane was blocked with 5% milk for 1 h, incubated with the relevant primary antibodies at 4°C overnight, and was then incubated with horseradish-peroxidase-conjugated secondary antibodies (1:2000) at room temperature for 1 h. An ECL Kit (MilliporeSigma) was used to detect the immunoreactive bands. The primary antibodies: Carabin (1:500) (Cat#PA5-20394, Thermo Scientific), Calcineurin (1:1000) (Cat#2614S, Cell Signaling, Danvers, MA, USA), NFAT (1:1000) (Cat#5861S, Cell Signaling), Ras (1:1000) (Cat#3339S, Cell Signaling), ERK (1:1000) (Cat#4696S, Cell Signaling) and p-ERK (1:500) (Cat#8544S, Cell Signaling). Antibodies used in this study for WB were listed in **Supplementary Materials**.

### Statistical Analysis

All of the data are expressed as mean ± SEM. Comparisons between two groups were performed using an unpaired student’s t test and multiple groups were evaluated using one-way ANOVA followed by Bonferroni’s analysis (for data meeting homogeneity of variance) or Tamhane’s T2 analysis (for data demonstrating heteroscedasticity). Statistical analysis was performed using Prism8 software (GraphPad Software, La Jolla, CA, USA). In general, P values < 0.05 were considered statistically significant.

## Data Availability Statement

The raw data supporting the conclusions of this article will be made available by the authors, without undue reservation.

## Ethics Statement

The animal study was reviewed and approved by Nanjing Medical University.

## Author Contributions

XN and LL designed research. XN, XW, X-XZ and J-HL performed research. X-HN and XW analyzed data. X-YY and LL gave suggestion. X-YY and LL reversed the paper. X-HN wrote the paper. All authors contributed to the article and approved the submitted version.

## Funding

This work was supported by grants from the fellowship of China National Postdoctoral Program for Innovative Talents (BX20200397) and the fellowship of China National Postdoctoral Science Foundation (2020M683086). This work was also supported by grants from the Research Unit of Liver Transplantation and Transplant Immunology of Chinese Academy of Medical Sciences (2019-I2M-5-035).

## Conflict of Interest

The authors declare that the research was conducted in the absence of any commercial or financial relationships that could be construed as a potential conflict of interest.

## Publisher’s Note

All claims expressed in this article are solely those of the authors and do not necessarily represent those of their affiliated organizations, or those of the publisher, the editors and the reviewers. Any product that may be evaluated in this article, or claim that may be made by its manufacturer, is not guaranteed or endorsed by the publisher.

## Supplementary Material

Supplementary Figure 1 Knockdown of Carabin in myeloid cells does not affect I/R-induced macrophage infiltration. **(A, B)** F4/80-positive cells in liver tissue were detected by immunohistochemistry. Scale bar, 100 µm. Statistical analyses of the number of F4/80-positive cells. Data are presented as the mean ± SEM (n = 5 mice/group, 3-5 fields were quantified). ns, not significant.
