# Supplementary figures and images for: Carabin Deficiency Aggravates Hepatic Ischemia-Reperfusion Injury Through Promoting Neutrophil Trafficking via Ras and Calcineurin Signaling
Source: Front Immunol. 2022 Feb 21;13:773291. doi: 10.3389/fimmu.2022.773291 (PMC8898835; doi:10.3389/fimmu.2022.773291)

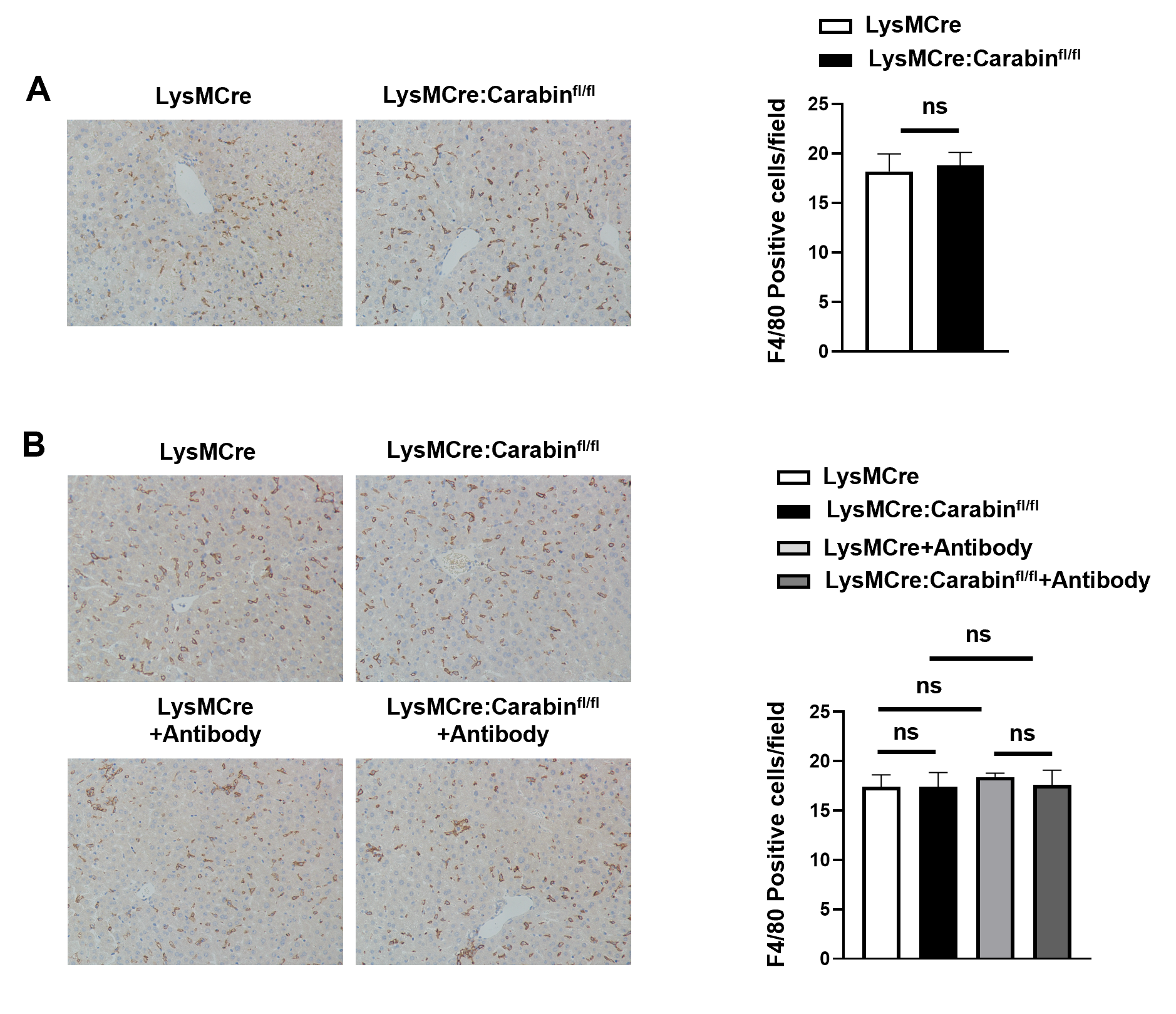

Supplement: Supplementary Figure 1 — Knockdown of Carabin in myeloid cells does not affect I/R-induced macrophage infiltration. (A, B) F4/80-positive cells in liver tissue were detected by immunohistochemistry. Scale bar, 100 µm. Statistical analyses of the number of F4/80-positive cells. Data are presented as the mean ± SEM (n = 5 mice/group, 3-5 fields were quantified). ns, not significant. [file Image_1.tif]
